# Supplementary material for: Dependence of cell fate potential and cadherin switching on the coordinate within the primitive streak during differentiation of human pluripotent stem cells
Source: Development. 2026 Apr 14;153(7):dev204807. doi: 10.1242/dev.204807 (PMC13120680; doi:10.1242/dev.204807)
Supplement: Supplementary information [file develop-153-204807-s1.pdf]

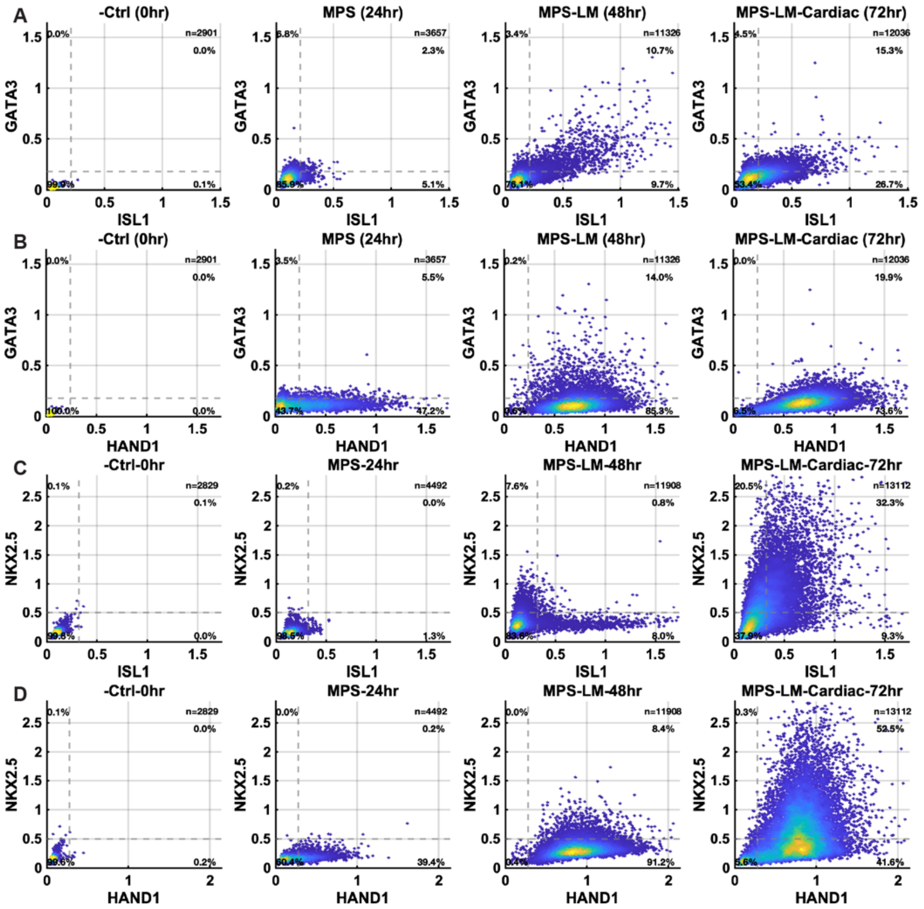

Fig. S1. Cells in LM conditions contain a subset of amnion cells and are competent to generate cardiac progenitors. Co-expression scatter plots of GATA3 and ISL1 (**A**), GATA3 and HAND1 (**B**), NKX2.5 and ISL1 (**C**), and NKX2.5 and HAND1 (**D**) for cells after 0hr, 24hr, 48hr, and 72hr MPS-LM-Cardiac treatment (0-24hr in MPS treatment; 24-48hr in LM treatment; 48-72hr in cardiac treatment). – Ctrl: negative control that is maintained in mTeSR Plus, fixed at 0hr. GATA3: amnion marker. NKX2.5: cardiac marker. HAND1 and ISL1: LM fate marker. (2 independent experiments performed. 6 images per condition.) Statistical comparisons were performed on per-image medians from two independent experiments using Welch's two-sample *t*-test with Bonferroni correction. Asterisks denote adjusted significance ($p < 0.05$; *$p < 0.01$; **$p < 0.001$; ***$p < 0.0001$), relative to the standard treatment group.

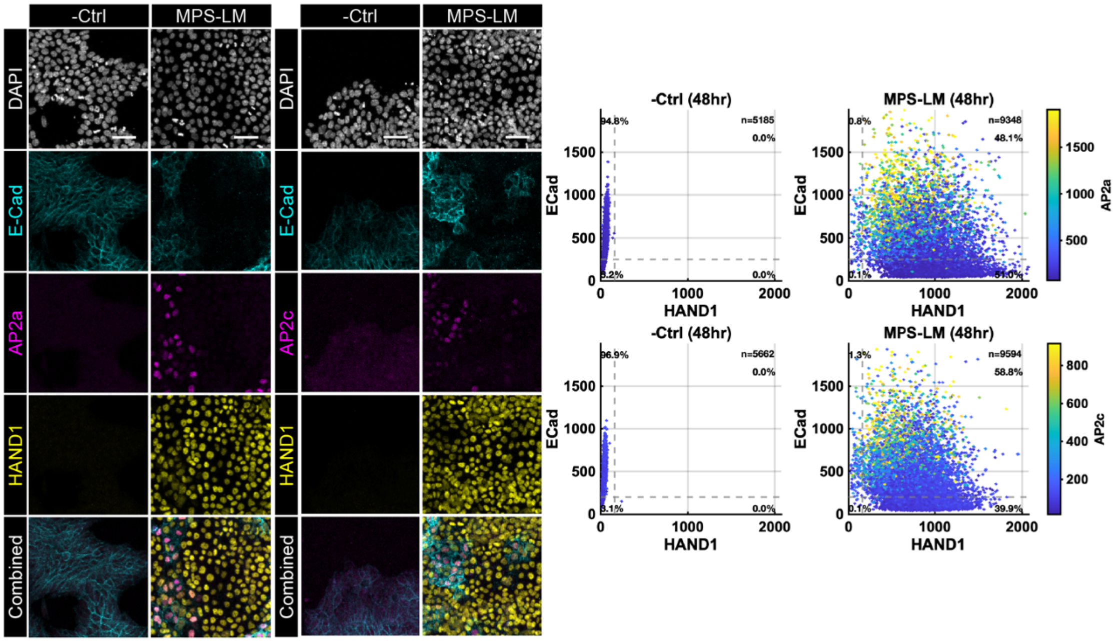

Fig. S2. MPS-LM treatment induces amnion-like cells co-expressing AP2a, AP2c, and HAND. Example confocal immunofluorescent images of cells stained for E-Cad, AP2a, AP2c, and HAND1 for the indicated treatments. Scale Bars: 50 µm. Scatter plots show co-expression of E-Cad and HAND1 at the single-cell level, with color indicating relative expression levels of AP2a or AP2c (see color bar). Each dot represents one cell (n = [the total number of cells per condition]). Dashed lines denote manually defined thresholds used for quadrant separation. Percentages indicate the proportion of cells in each quadrant. (2 independent experiments performed. 7 images per condition.)

Development • Supplementary information

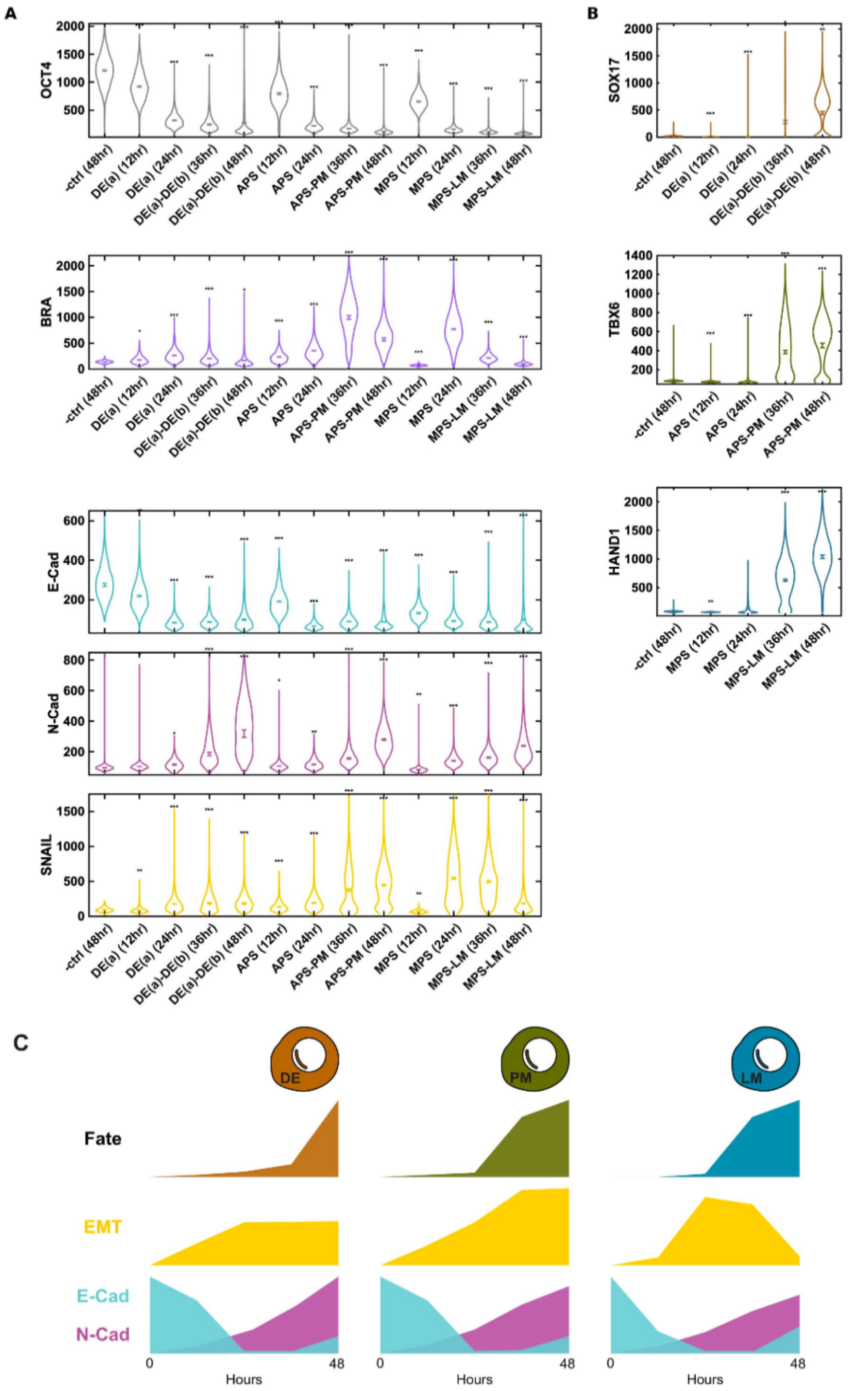

Fig. S3. Dynamics of fate commitment in LM, PM, and DE differentiation.

Quantification of nuclear and membrane marker expression of DE(a)-DE(b), APS-PM, and MPS-LM treated cells at 12hr, 24hr, 36hr, and 48hr after induction start, using confocal microscopy. - ctrl: negative control maintained in mTeSR Plus. **A.** OCT4: pluripotency marker. BRA: PS transient marker; E-Cad: E- Cadherin. N-Cad: N-Cadherin. Snail: EMT marker. **B.** SOX17: DE fate marker. TBX6: PM fate marker. HAND1: LM fate marker. (2 independent experiments performed. 7 images per condition.) **C**. Schematic comparing timing of fate marker expression, cadherin switching, and EMT during differentiation protocols. Statistical comparisons were performed on per-image medians from two independent experiments using Welch's two-sample t-test with Bonferroni correction. Asterisks denote adjusted significance ($p < 0.05$; *$p < 0.01$; **$p < 0.001$; ***$p < 0.0001$), relative to the standard treatment group.

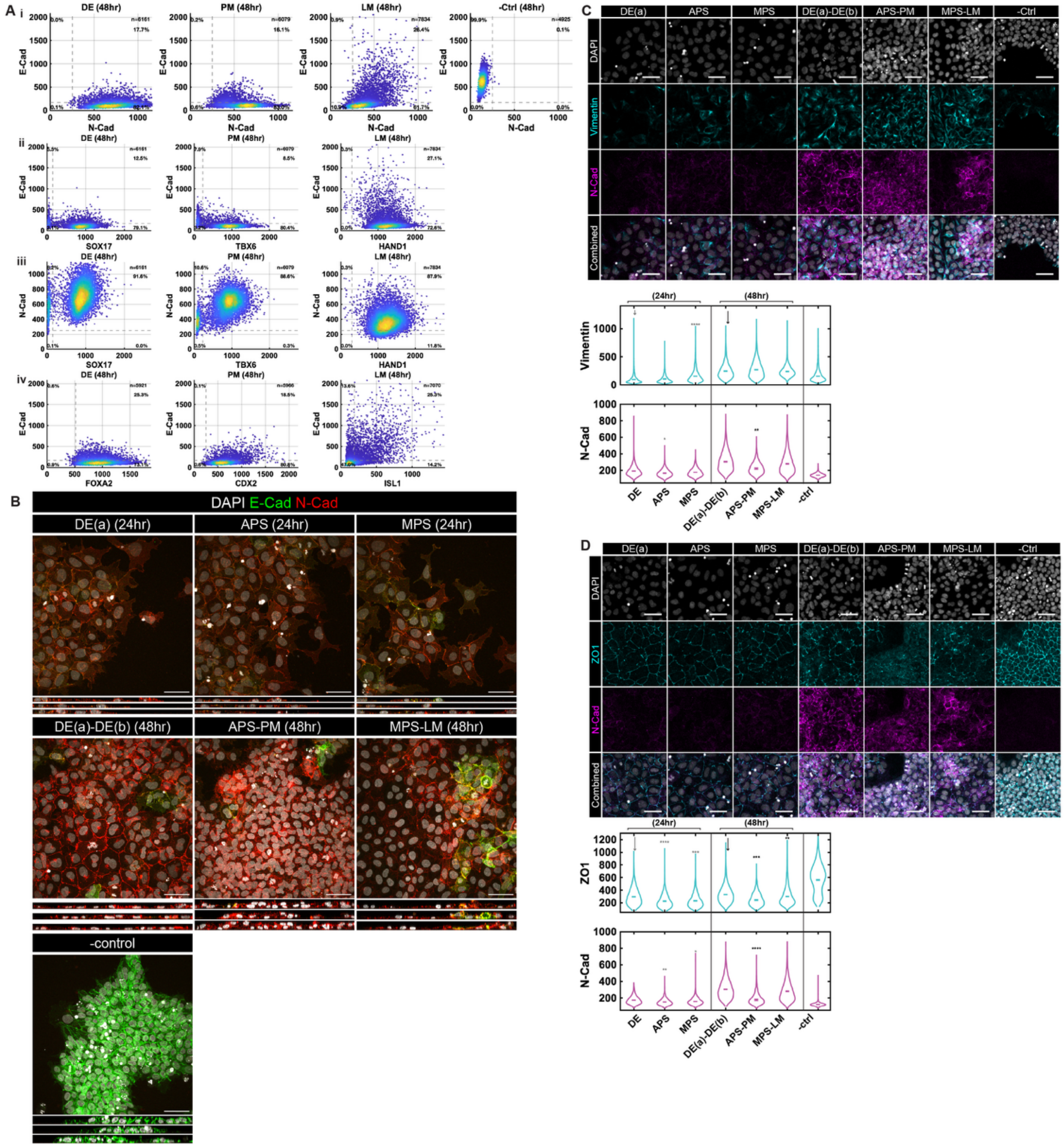

Fig. S4. Correlation between cadherins and fate commitment in mesendodermal differentiation. A. Co-expression scatter plots of E-Cad and N-Cad (**i**), E-Cad and fate markers (**ii** and **iv**), and N-Cad and fate markers (**iii**) for DE, PM and LM cells after 48hr treatment. – Ctrl: negative control that is maintained in mTeSR Plus. SOX17 and FOXA2: DE fate marker. TBX6 and CDX2: PM fate

marker. HAND1 and ISL1: LM fate marker. (2 independent experiments performed. 6 images per condition.) **B**. Example confocal immunofluorescent images of high magnification (40x) and orthogonal view of DE(a), APS, MPS, DE(a)-DE(b), APS-PM, MPS-LM, and epithelial control cells (maintained in mTeSR Plus) immunostained with E-Cad, N-Cad, and DAPI. Scale Bars: 50 µm. **C and D**. Example confocal immunofluorescent images and and quantification based on fluorescent intensity of Vimentin (**C**) and ZO-1 (**D**) for DE(a), APS, MPS, DE(a)-DE(b), APS-PM, MPS-LM, and epithelial control cells (maintained in mTeSR Plus). Scale Bars: 50 µm. (2 independent experiments performed. 7 images per condition.) Statistical comparisons were performed on per-image medians from two independent experiments using Welch's two-sample *t*-test with Bonferroni correction. Asterisks denote adjusted significance ($p < 0.05$; *$p < 0.01$; **$p < 0.001$; ***$p < 0.0001$), relative to the standard treatment group.

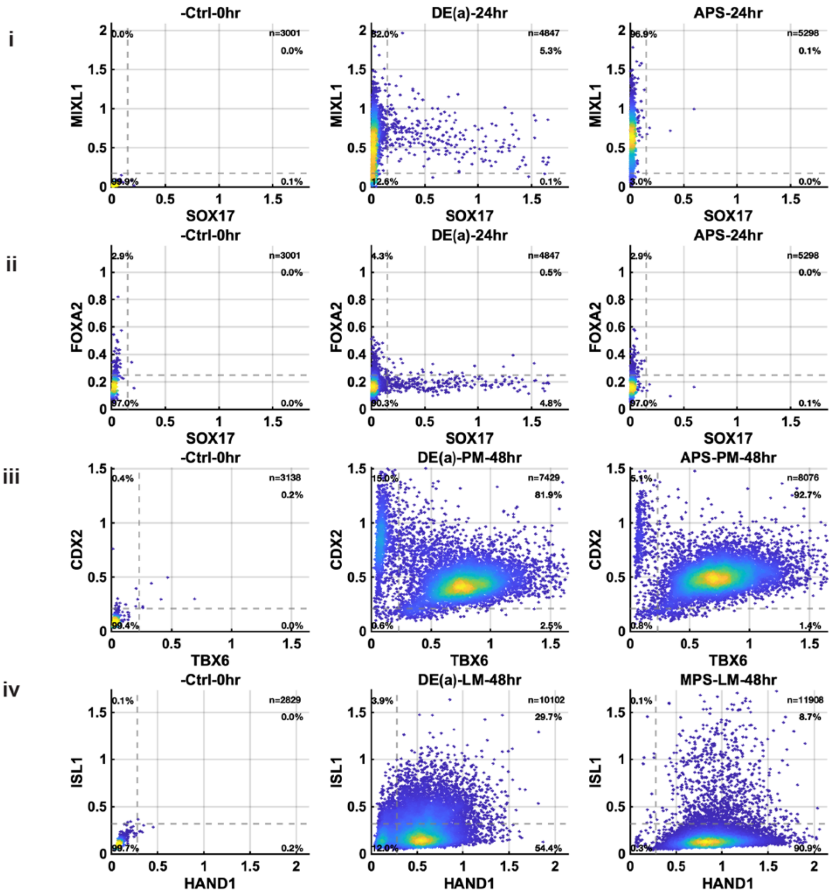

Fig. S5. Cells treated with DE(a) are competent to generate DE, PM, and LM. Co-expression scatter plots of (i) MIXL1 and SOX17 for DE(a), and APS cells after 24hr induction; (ii) FOXA2 and SOX17 for DE(a)-DE(b), and APS-DE(b) cells after 48hr induction; (iii) CDX2 and TBX6 for DE(a)-PM, and APS-PM cells after 48hr induction; (iv) ISL1 and HAND1 for DE(a)-LM, and MPS-LM cells after 48hr induction. Each dot represents one cell (n = [the total number of cells per condition]); color indicates density of overlapping points. Dashed lines denote manually defined thresholds used for quadrant separation. Percentages indicate the proportion of cells in each quadrant. (2 independent experiments performed. 6 images per condition.)

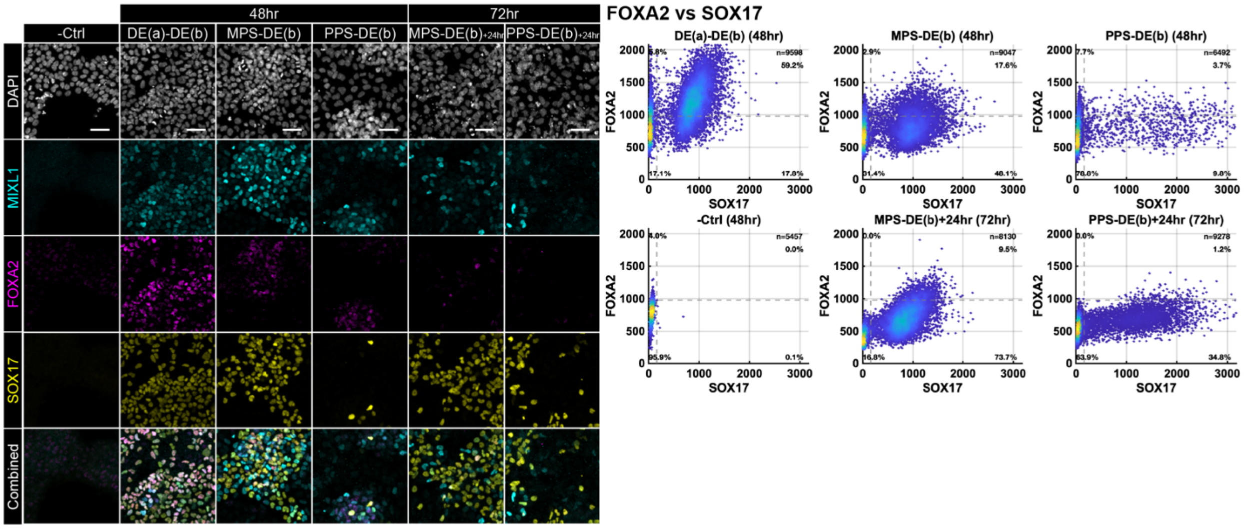

Fig. S6. MPS and PPS lose DE potency despite extended induction. Example confocal immunofluorescent images immunostained for MIXL1, FOXA2, and SOX17 for the indicated. Scale Bars: 50 μm. Scatter plots show co-expression of FOXA2 and SOX17 at the single-cell level. Each dot represents one cell (n = [the total number of cells per condition]); color indicates density of overlapping points. Dashed lines denote manually defined thresholds used for quadrant separation. Percentages indicate the proportion of cells in each quadrant. (2 independent experiments performed. 7 images per condition.)

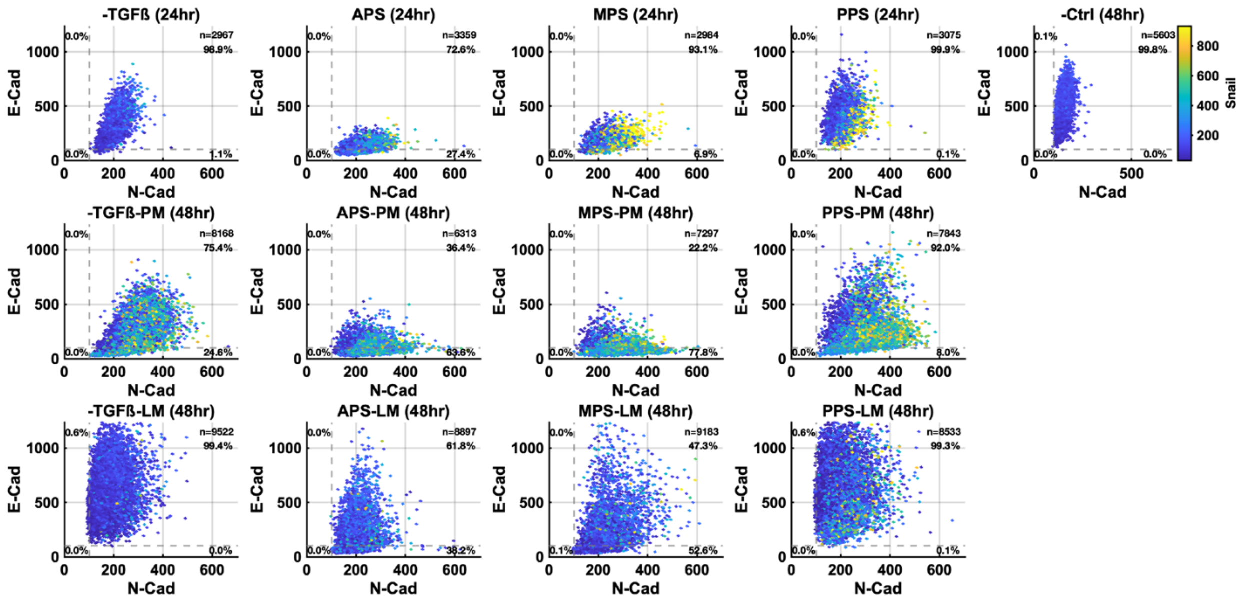

Fig. S7. Snail, E-cad, and N-cad expression are not correlated. Scatter plots show co-expression of E-cadherin and N-cadherin for cells treated by -TGFβ, APS, MPS or PPS and fixed by 24hr, along with negative control group (-Ctrl, maintained in mTeSR Plus and fixed at 0hr) and for the four PS treated groups further induced towards PM or LM. at the single-cell level, with color indicating relative expression levels of Snail (see color bar). Each dot represents one cell (n = [the total number of cells per condition]). Dashed lines denote manually defined thresholds used for quadrant separation. Percentages indicate the proportion of cells in each quadrant. (2 independent experiments performed. 8 images per condition.)

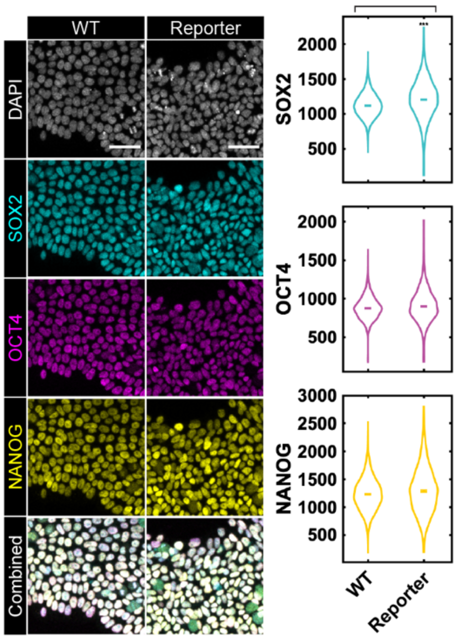

Fig. S8. Validation of ESI017 CAAX:mCerulean cell line. Example confocal immunofluorescent images and quantification based on fluorescent intensity of pluripotency markers, SOX2, OCT4, and NANOG for ESI017 wildtype (WT), and CAAX:mCerulean (maintained in mTeSR Plus). DAPI: nuclear marker. Scale Bars: 50 µm. (2 independent experiments performed. 7 images per condition.) Statistical comparisons were performed on per-image medians from two independent experiments using Welch's two-sample *t*-test with Bonferroni correction. Asterisks denote adjusted significance ($p < 0.05$; *$p < 0.01$; **$p < 0.001$; ***$p < 0.0001$), relative to the standard treatment group.

Development • Supplementary information

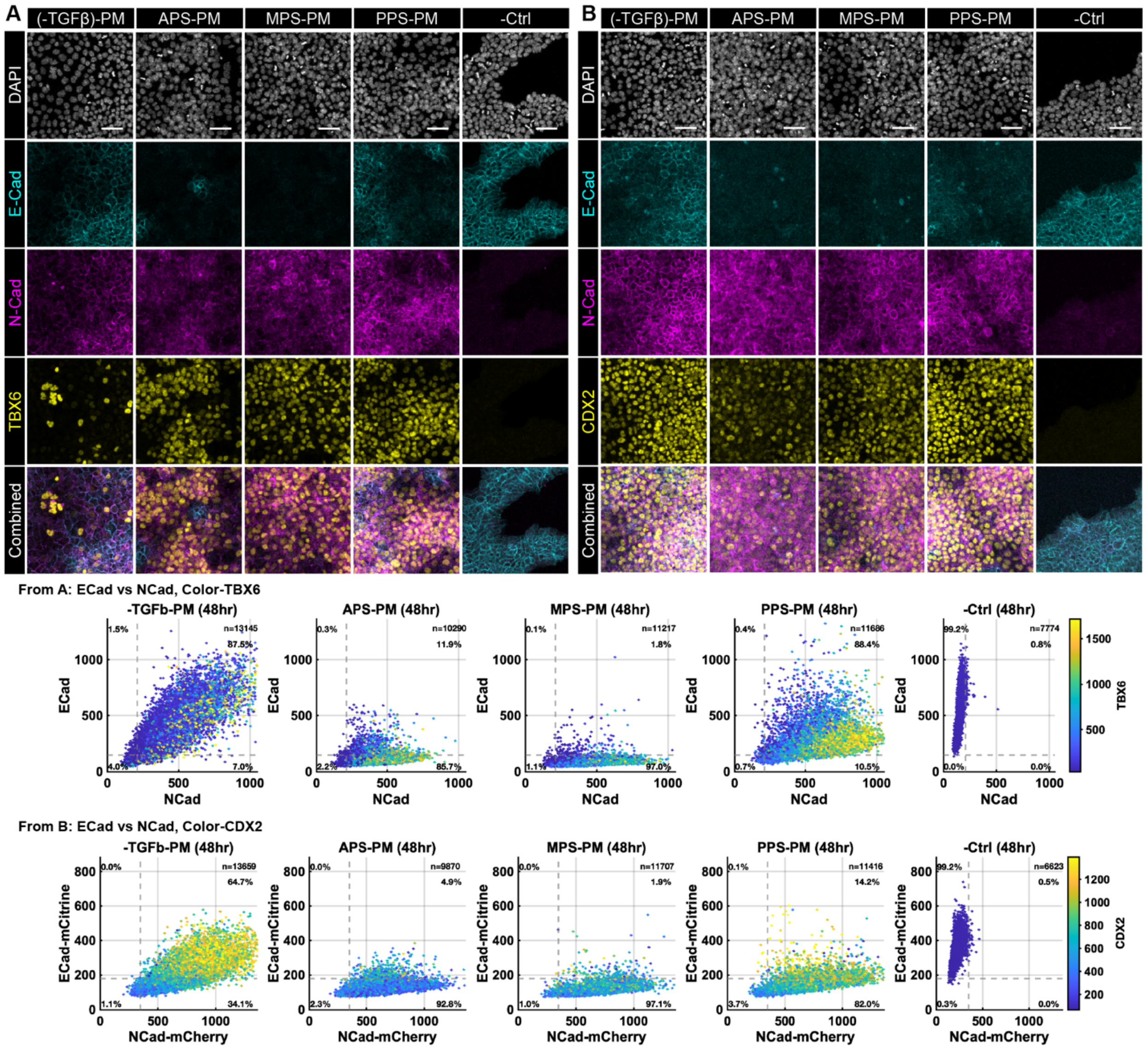

Fig. S9. E- to N-cadherin dynamics in relation to paraxial mesoderm fate markers. Example confocal immunofluorescent images of cells stained for E-cadherin, N-cadherin, and PM fate markers (CDX2 and TBX6 (**A** and **B**)) for the indicated treatments. Either Wild type ESI017 or ESI017- CDH1:mCitrine-CDH2:mCherry-CAAX:mCerulean reporter cell lines were used for each treatment. Scale Bars: 50 µm. Scatter plots show co-expression of E-cadherin and N-cadherin at the single-cell level, with color indicating relative expression levels of CDX2 or TBX6 (see color bar). Each dot represents one cell (n = [the total number of cells per condition]). Dashed lines denote manually defined thresholds used for quadrant separation. Percentages indicate the proportion of cells in each quadrant. (2 independent experiments performed. 9 images per condition.)

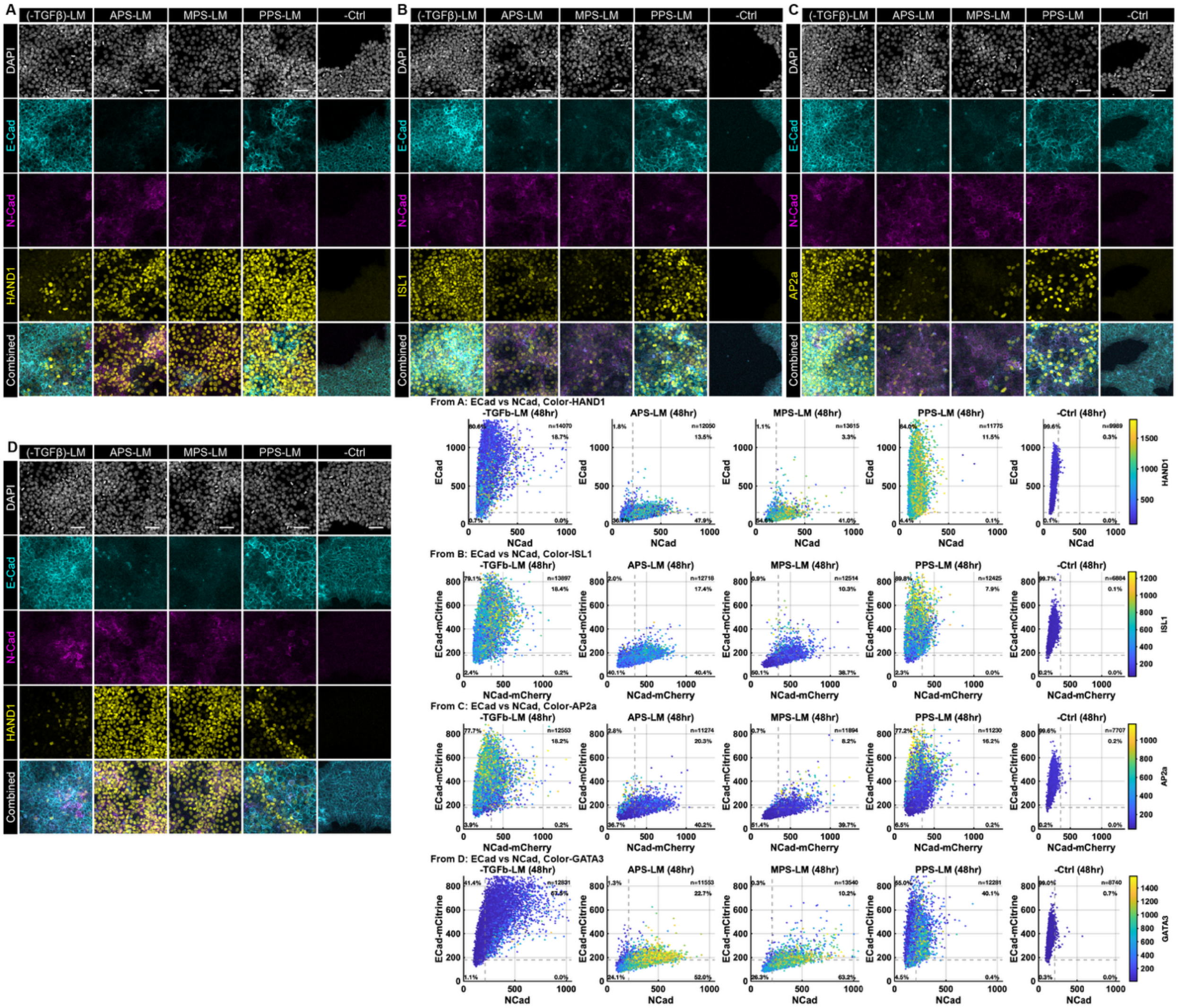

Fig. S10. E- to N-cadherin dynamics in relation to lateral mesoderm fate markers. Example confocal immunofluorescent images of cells stained for E-cadherin, N-cadherin, and LM fate markers (HAND1, ISL1, AP2a, and GATA3 (**A, B, C, D**)) for the indicated treatments. Either Wild type ESI017 or ESI017-CDH1:mCitrine-CDH2:mCherry-CAAX:mCerulean reporter cell lines were used for each treatment. Scale Bars: 50 µm. Scatter plots show co-expression of E-cadherin and N-cadherin at the single-cell level, with color indicating relative expression levels of HAND1, ISL1 or AP2a (see color bar). Each dot represents one cell (n = [the total number of cells per condition]). Dashed lines denote manually defined thresholds used for quadrant separation. Percentages indicate the proportion of cells in each quadrant. (2 independent experiments performed. 9 images per condition.)

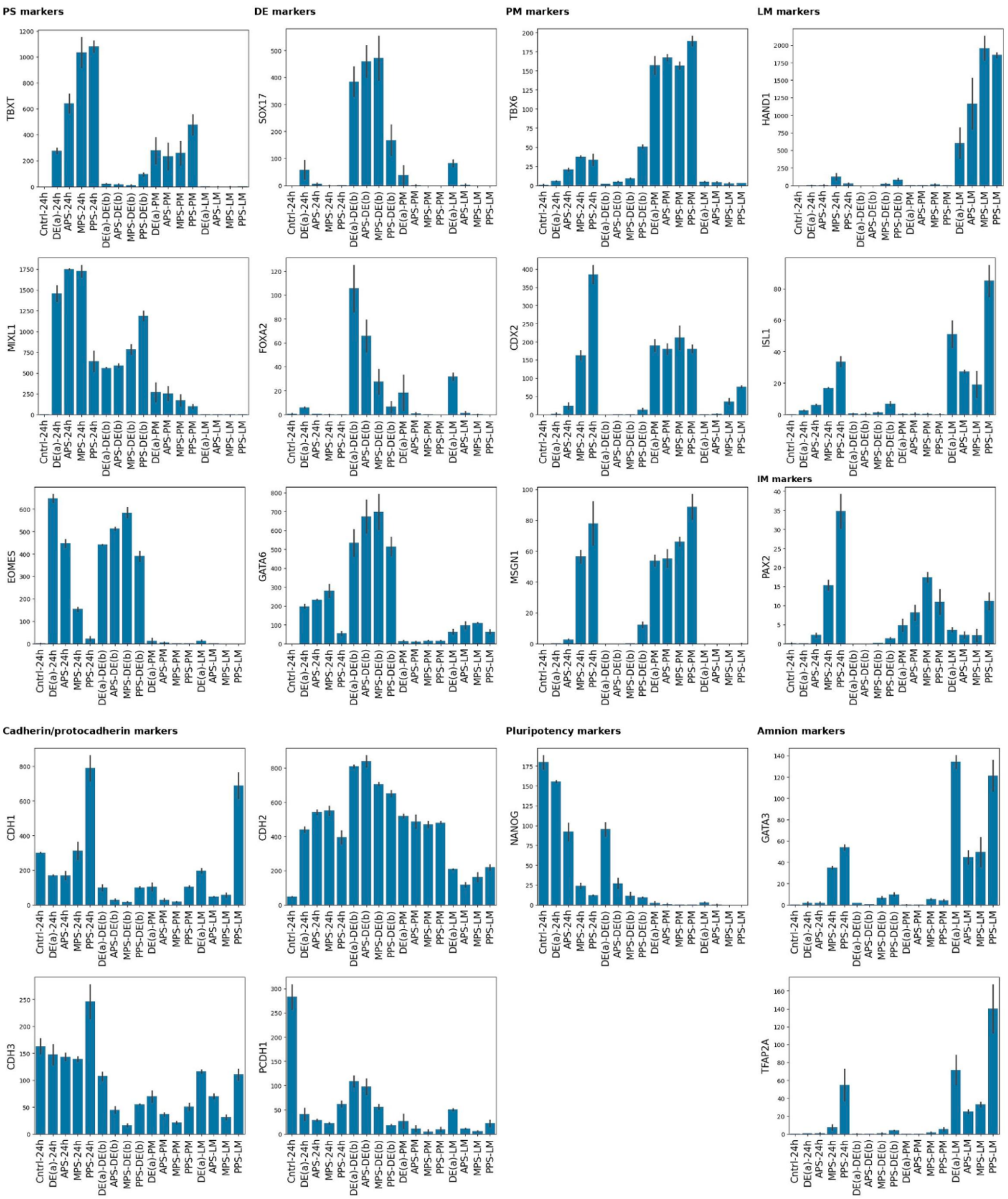

Fig. S11. Extended gene expression analysis from RNA sequencing data.

Bar plots showing transcript levels (TPM) of representative genes under the indicated treatments. Genes include pluripotency marker (NANOG), PS markers (MIXL1, EOMES, TBXT), DE markers (GATA6, FOXA2, SOX17), PM markers (TBX6, MSGN1, CDX2), LM markers (ISL1, HAND1), IM marker (PAX2), Amnion markers (GATA3, TFAP2A), and Cadherin and Protocadherin markers (CDH1, CDH2, CDH3, PCDH1). Expression values are presented as transcripts per million (TPM). Error bars represent standard deviation across biological replicates (N=2).

Development • Supplementary information

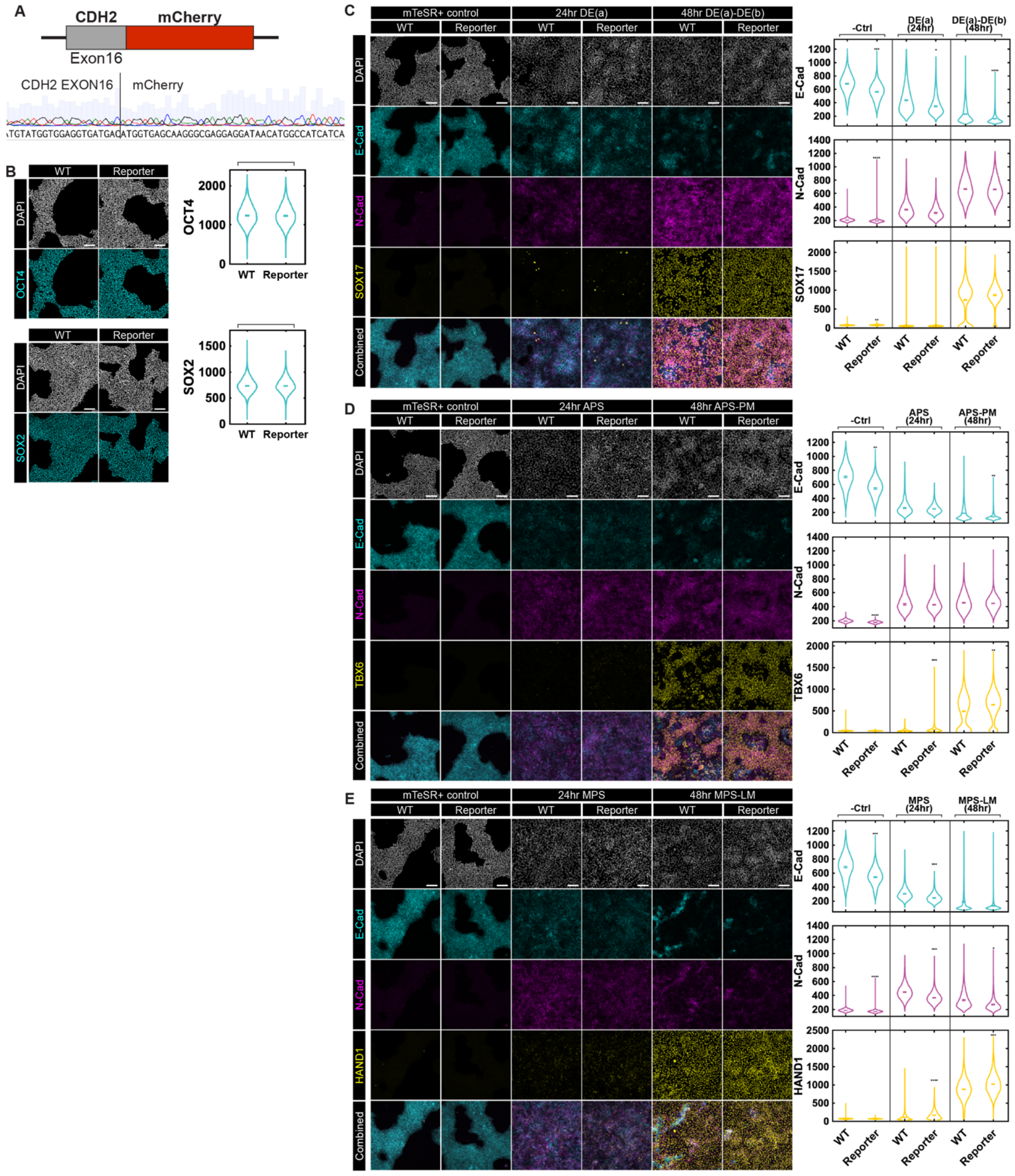

Fig. S12. Characterization of the E-Cad/N-Cad dual reporter line. A. Sequencing result of modified CDH2 locus showing connection of CDH2 Exon 16 and mCherry coding sequence. The base cell line is ESI-017 CDH1:mCitrine. CAAX-mCerulean was also inserted to make ESI017 CDH1:mCitrine-CDH2:

Development • Supplementary information

mCherry-CAAX:mCeruleanReporter cell line. **B**. Pluripotency validation of ESI017- CDH1:mCitrine-CDH2:mCherry-CAAX:mCeruleancell line. Example images and quantification based on fluorescent intensity of pluripotency markers, OCT4, and SOX2, for ESI017 wildtype (WT), and ESI017-CDH1:mCitrine-CDH2:mCherry-CAAX:mCerulean (maintained in mTeSR Plus). Scale Bars: 50 μm. **C**, **D** and **E**. Example confocal immunofluorescent images and quantification based on fluorescent intensity of E-Cad, N-Cad and fate markersfor ESI017-CDH1:mCitrine-CDH2:mCherry-CAAX:mCeruleanReporter cell line and wild type (WT) ESI0-17 underwent DE(a)-DE(b) (SOX17) (**C**), APS-PM (TBX6) (**D**), and MPS-LM (HAND1) (**E**) treatment, respectively. DAPI: nuclear marker. Scale Bars: 50 μm. (2 independent experiments performed. 7 images per condition.) Statistical comparisons were performed on per-image medians from two independent experiments using Welch's two-sample *t*-test with Bonferroni correction. Asterisks denote adjusted significance ($p < 0.05$; *$p < 0.01$; **$p < 0.001$; ***$p < 0.0001$), relative to the standard treatment group.

Development • Supplementary information

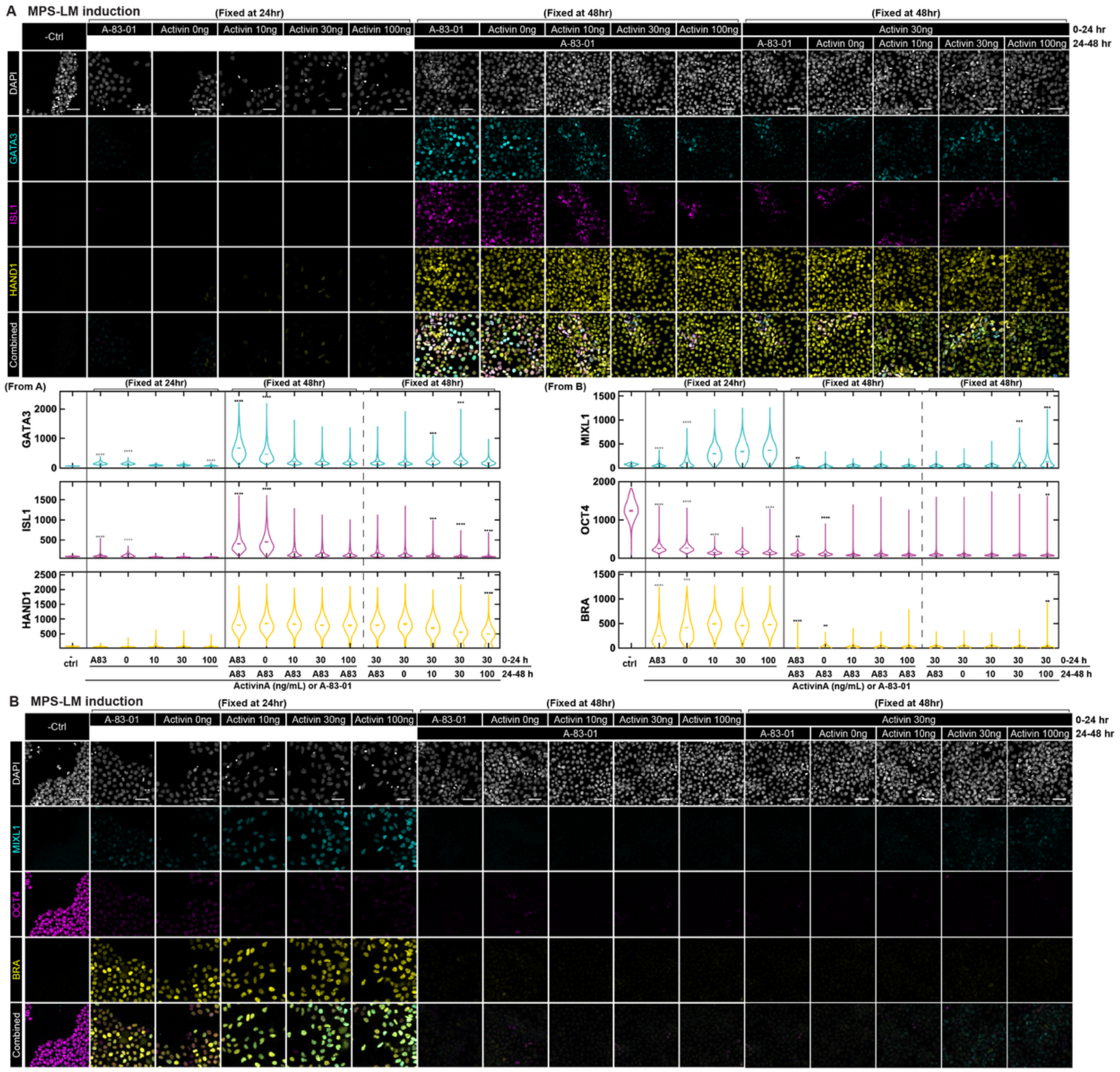

Fig. S13. Effects of varying Activin signaling levels on LM fate commitment. Example confocal immunofluorescent images and quantification based on fluorescent intensity of GATA3, ISL1, and HAND1 (**A**), and MIXL1, OCT4 and BRA (**B**) for cells underwent MPS induction with A-83-01 (1 μM) or various Activin A doses (0, 10, 30, and 100 ng/mL) and fixed by 24hr; for all the MPS induced cells continued with LM treatment (A-83-01 1 μM) and fixed by 48hr; and for cells underwent original MPS treatment (Activin A at 30 ng/mL), and switched to LM treatment with A-83-01 (1 μM) or various Activin A doses (0, 10, 30, and 100 ng/mL) and fixed by 48hr. (2 independent experiments performed. 7 images per condition.) Images from the standard differentiation condition at 48 hours (Activin 30ng/ml followed by A-83-01) are shown within each series (columns 10 and 12) for ease of comparison. Statistical comparisons were performed on per-image medians from two independent experiments using Welch's two-sample *t*-test with Bonferroni correction. Asterisks denote adjusted significance ($p < 0.05$; *$p < 0.01$; **$p < 0.001$; ***$p < 0.0001$), relative to the standard treatment group.

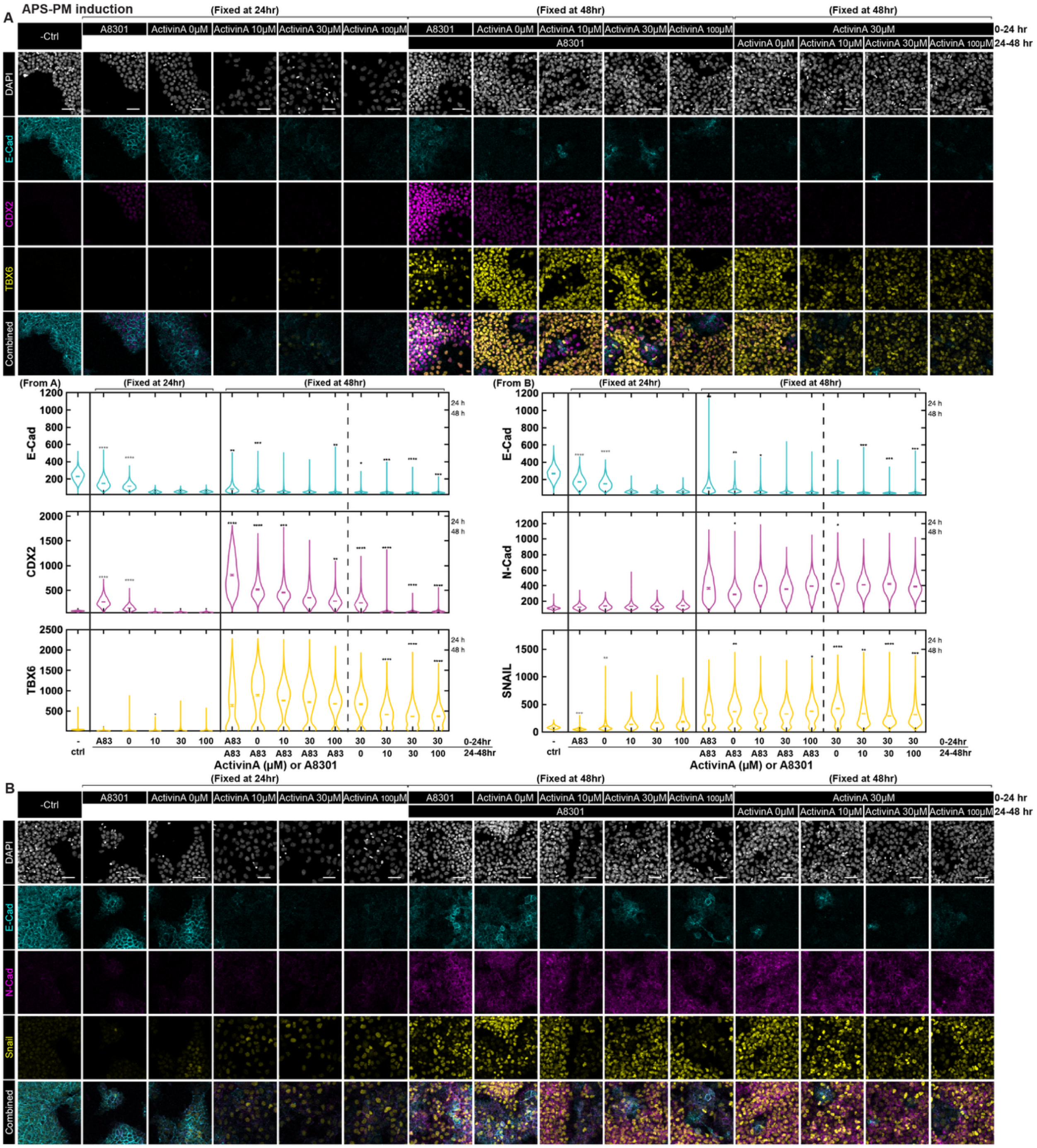

Fig. S14. Dose-dependent effects of Activin signaling on cadherin switching and paraxial mesoderm fate induction

Example confocal immunofluorescent images and quantification based on fluorescent intensity of E-Cad, TBX6, and CDX2 (**A**); E-Cad, N-Cad, and Snail (**B**) for cells that underwent APS or APS-PM induction with either A-83-01 (1 μM) or various Activin A doses (0, 10, 30, and 100 ng/mL) and fixed by 24hr. Scale Bars: 50 μm. In the violine plots, black bars indicate per-image means. Statistical comparisons were performed on per-image medians from two independent experiments using Welch's two-sample *t*-test with Bonferroni correction. Asterisks denote adjusted significance ($p < 0.05$; *$p < 0.01$; **$p < 0.001$; ***$p < 0.0001$), relative to the standard treatment group. (2 independent experiments performed. 7 images per condition.)

Development • Supplementary information

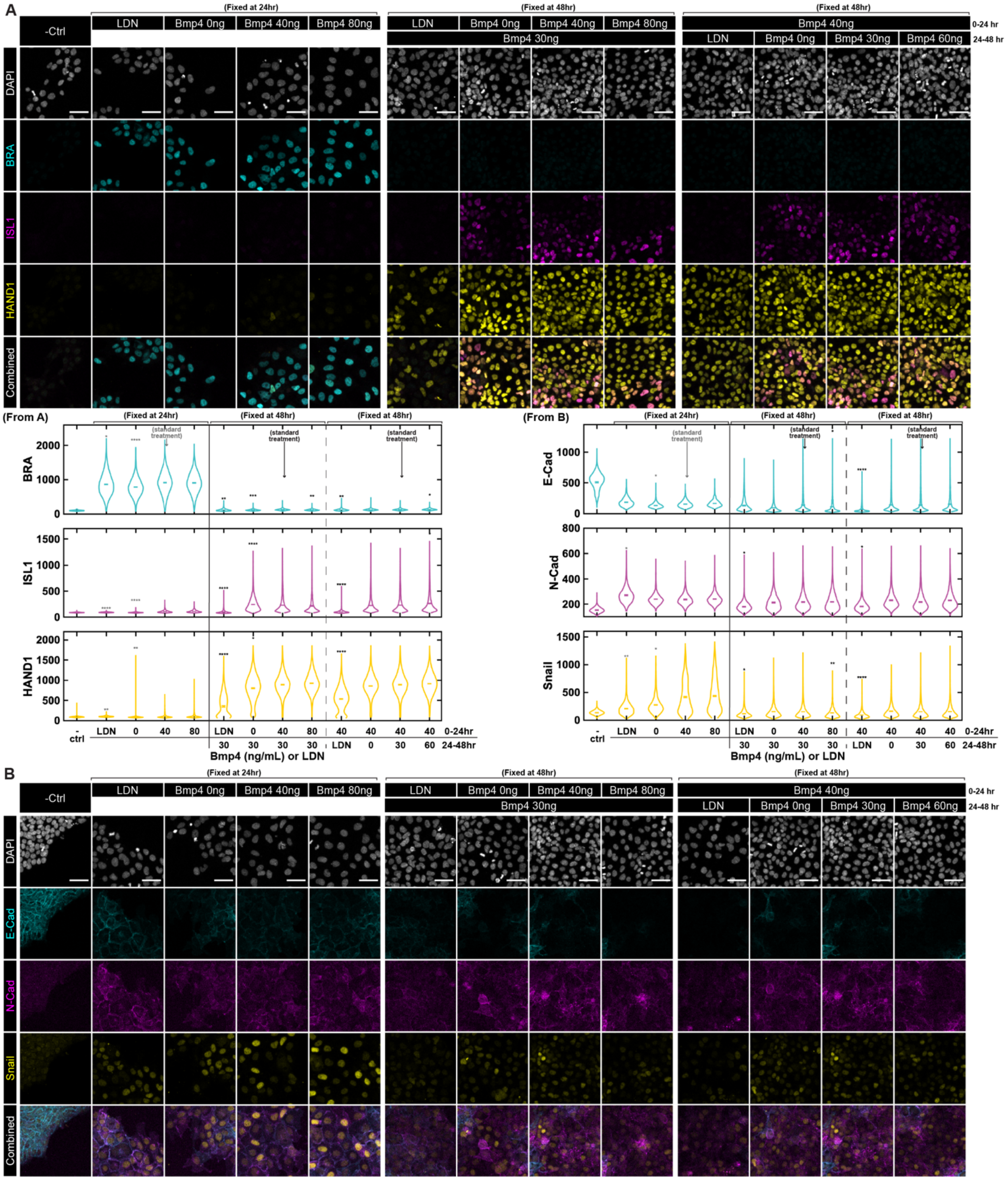

Development • Supplementary information

Fig. S15. Effects of varying BMP signaling levels on EMT and cadherin switching. Example confocal immunofluorescent images and quantification based on fluorescent intensity of BRA, ISL1, and HAND1 **(A)**, and E-CAD, N-Cad and Snail **(B)** for cells underwent MPS induction with LDN or various Bmp4 doses (0, 40, and 80 ng/mL) and fixed by 24hr; for all the MPS induced cells continued with LM treatment (Bmp4 at 30 ng/mL) and fixed by 48hr; and for cells underwent original MPS treatment (Bmp4 at 40 ng/mL), and switched to LM treatment with LDN or various Bmp4 doses (0, 30, and 60 ng/mL) and fixed by 48hr. (2 independent experiments performed. 8 images per condition.) Images from the standard differentiation condition at 48 hours (BMP4 40ng/ml followed by BMP4 30 ng/ml) are shown within each series (columns 8 and 12) for ease of comparison. Statistical comparisons were performed on per-image medians from two independent experiments using Welch's two-sample t-test with Bonferroni correction. Asterisks denote

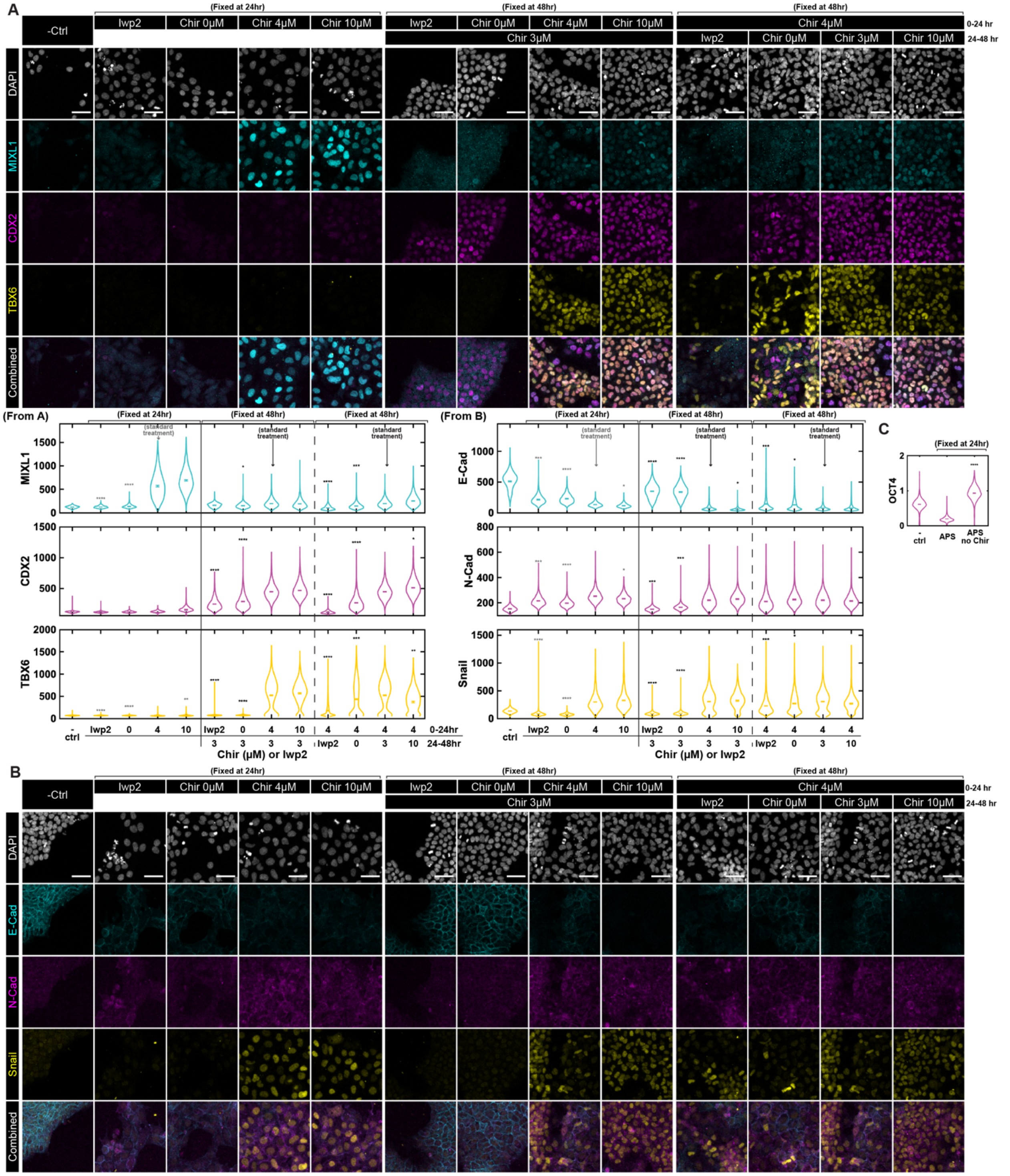

Fig. S16. Effects of varying WNT signaling levels on EMT and cadherin switching. Example confocal immunofluorescent images and quantification based on fluorescent intensity of MIXL1, CDX2, and TBX6 (**A**), and E-CAD, N-Cad and Snail (**B**) for cells underwent APS induction with IWP2 or various CHIR doses (0, 4, and 10 μM) and fixed by 24hr; for all the APS induced cells continued with PM treatment (CHIR at 3 μM) and fixed by 48hr; and for cells underwent original APS treatment (CHIR at 4 μM), and switched to PM treatment with IWP2 or various CHIR doses (0, 3, and 10 μM) and fixed by 48hr. **C.** Quantification based on inflorescent intensity of pluripotency marker, OCT4, for cells treated using mTeSR Plus (- Ctrl), APS, or APS with no CHIR. (2 independent experiments performed. 8 images per condition.) Images from the standard differentiation condition at 48 hours (CHIR 4μm followed by CHIR 3μm) are shown within each series (columns 8 and 12) for ease of comparison. This experiment was performed simultaneously with that shown in Fig. S15 and the images from the negative control are shown here as well. Statistical comparisons were performed on per-image medians from two independent experiments using Welch's two-sample t-test with Bonferroni correction. Asterisks denote adjusted significance *(p* < 0.05; **p* < 0.01; ***p* < 0.001; ****p* < 0.0001), relative to the standard treatment group.

Development • Supplementary information

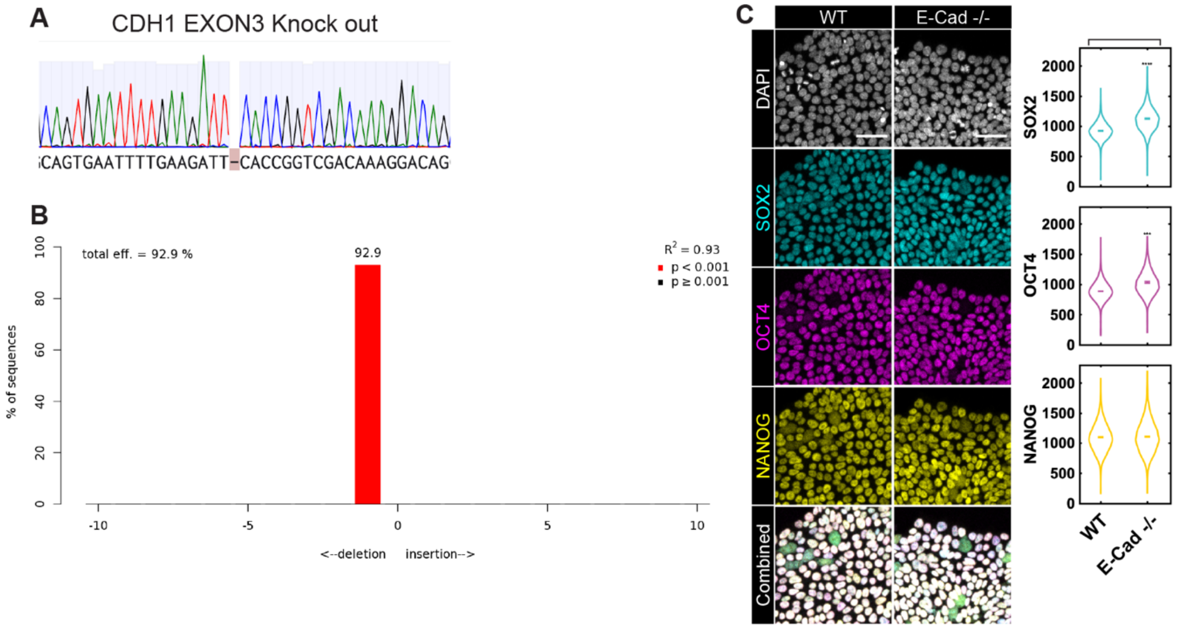

Fig. S17. Validation of CDH1 (E-Cadherin) knock-out. A. ESI017-CDH1 $^{-/-}$ was verified by sequencing, where the same deletion of 1 bp causing open reading frame (ORF) shifts for both alleles. **B**. A comprehensive profile of all insertions and deletions in the ESI017-CDH1 $^{-/-}$ sample by TIDE analysis (Brinkman et al., 2014). **C**. Pluripotency validation of ESI017- CDH1 $^{-/-}$ cell line. Example confocal immunofluorescent images and quantification based on fluorescent intensity of pluripotency markers, SOX2, OCT4, and NANOG for ESI017 wildtype (WT), and CDH1 $^{-/-}$ (maintained in mTeSR Plus). DAPI: nuclear marker. Scale Bars: 50 µm. (2 independent experiments performed. 7 images per condition.) Statistical comparisons were performed on per-image medians from two independent experiments using Welch's two-sample *t*-test with Bonferroni correction. Asterisks denote adjusted significance ($p < 0.05$; *$p < 0.01$; **$p < 0.001$; ***$p < 0.0001$), relative to the standard treatment group.

Development • Supplementary information

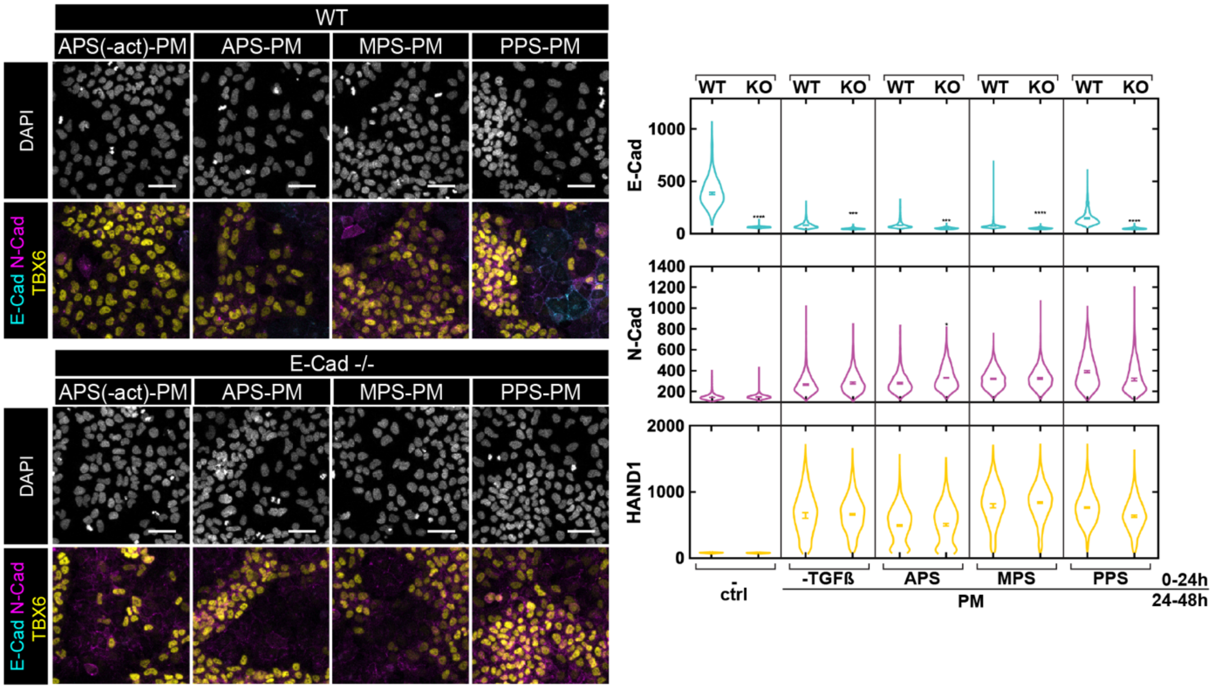

Fig. S18. Loss of E-CAD did not affect fate outcomes during PM differentiation. Example confocal microscopic images and quantification of E-Cad, N-Cad, and fate markers for WT and E-Cad $^{-/-}$ cells treated by -TGFβ, APS, MPS or PPS (0 – 24 hr), further induced to PM (24 – 48 hr; fate marker – TBX6), and fixed at 48 hr. Scale Bars: 50 µm. – Ctrl: negative control that is maintained in mTeSR Plus. (2 independent experiments performed. 5 images per condition.) Statistical comparisons were performed on per-image medians from two independent experiments using Welch's two-sample *t*- test with Bonferroni correction. Asterisks denote adjusted significance ($p < 0.05$; *$p < 0.01$; **$p < 0.001$; ***$p < 0.0001$), relative to the standard treatment group.

Development • Supplementary information

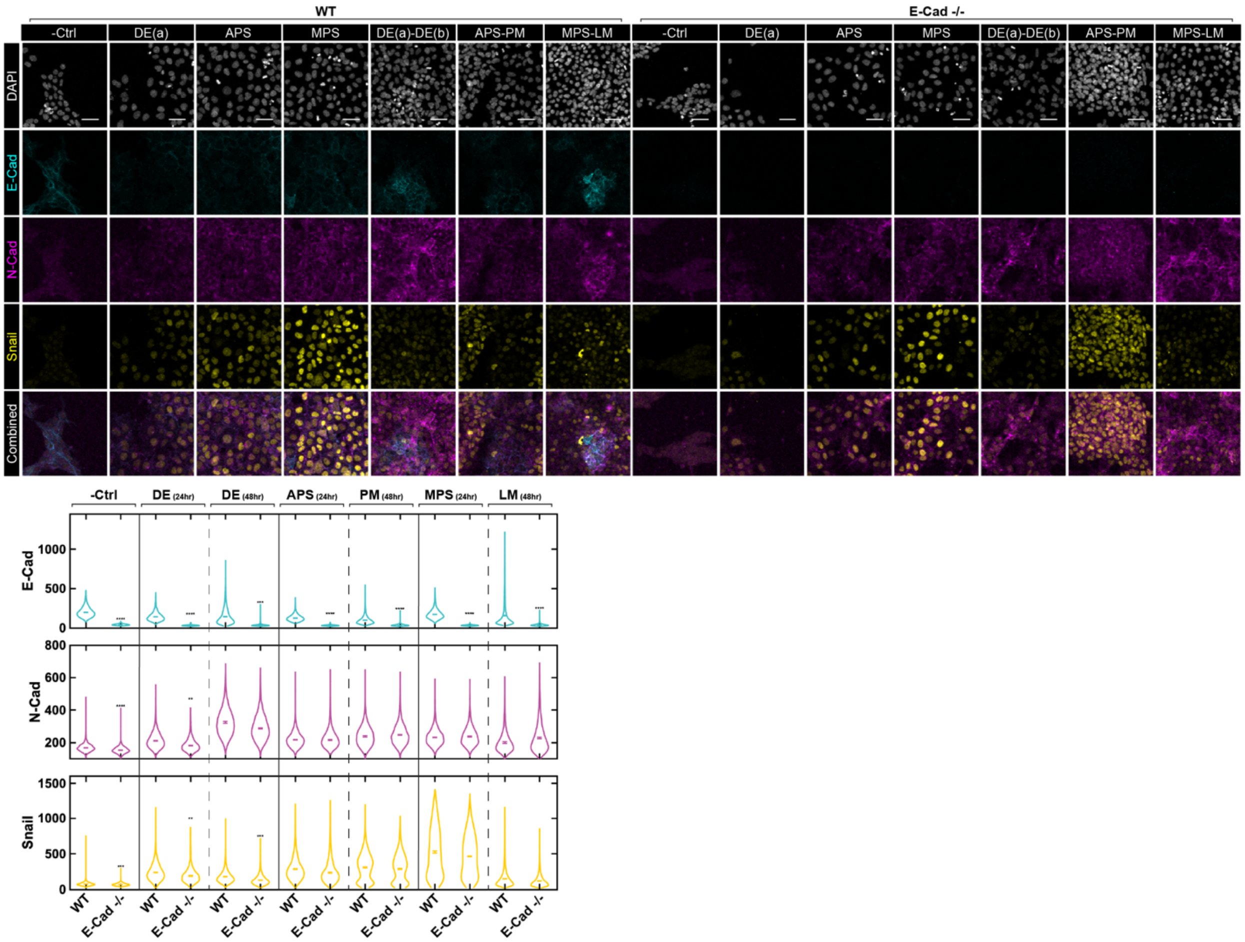

Fig. S19. Effects of loss of E-CAD on N-CAD and Snail expression during differentiation. Example confocal immunofluorescent images and quantification based on fluorescent intensity of E-Cad, N-Cad, and Snail for ESI017 wildtype (WT), and CDH1 $^{-/-}$ induced towards DE(a), APS, and MPS (fixed by 24hr), and further induced towards DE(b), PM and LM. DAPI: nuclear marker. Scale Bars: 50 µm. (2 independent experiments performed. 8 images per condition.) Statistical comparisons were performed on per-image medians from two independent experiments using Welch's two-sample *t*-test with Bonferroni correction. Asterisks denote adjusted significance ($p < 0.05$; *$p < 0.01$; **$p < 0.001$; ***$p < 0.0001$), relative to the standard treatment group.

Development • Supplementary information

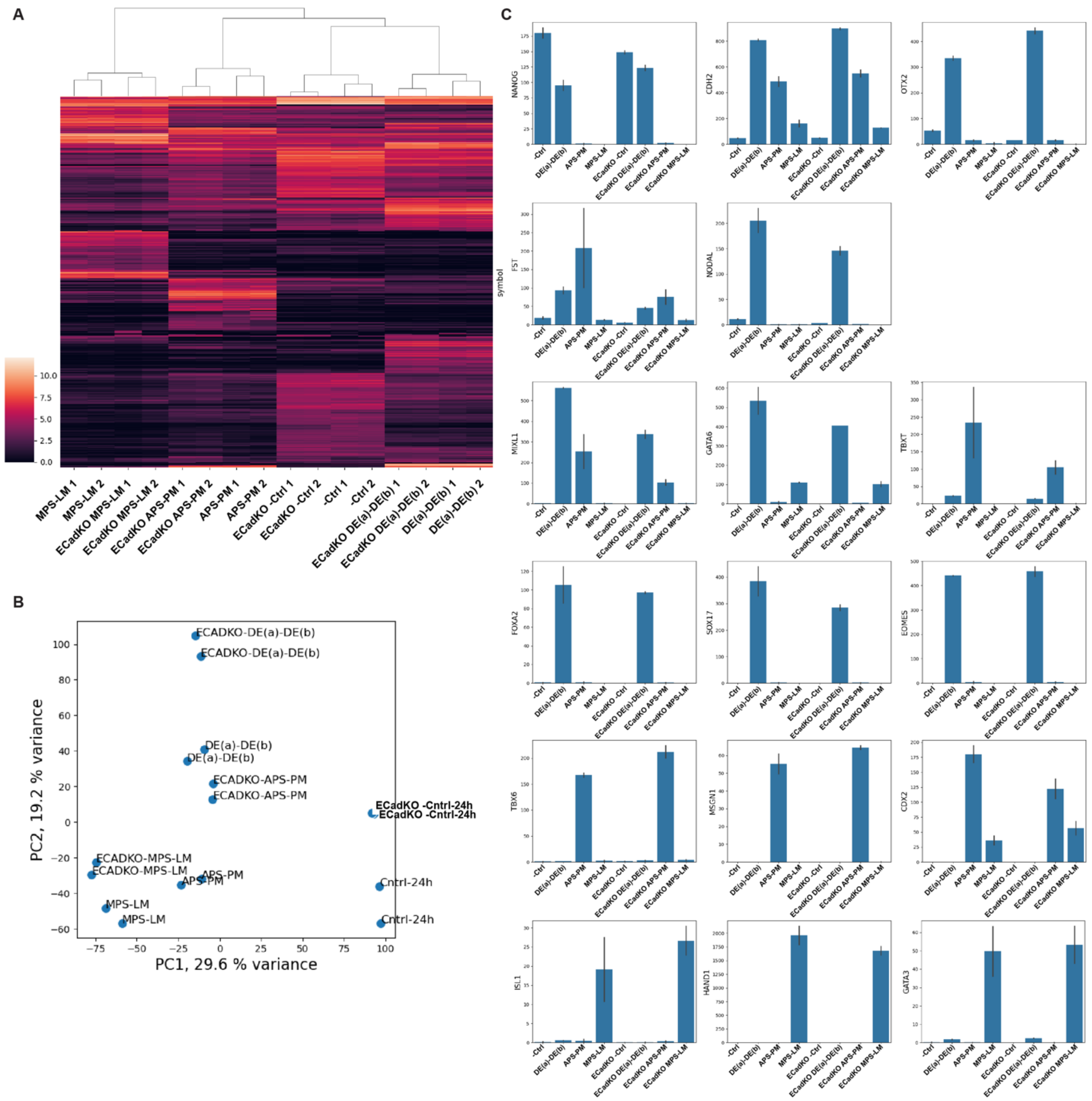

Fig. S20. Transcriptomic profiling of E-cadherin knockout and wild-type cells under DE, PM and LM induction. A. Heatmap of differentially expressed genes across wild-type ESI017 and E-cadherin knockout cells under the indicated treatments. Data are presented as log2(TPM+1) from bulk RNA-seq, with hierarchical clustering separating treatment groups. **B**. Principal component analysis (PCA) of transcriptomes showing clustering by genotype and treatment. **C**. Bar plots showing expression of selected marker genes associated with pluripotency, PS, DE, PM, LM and EMT. Expression values are presented as transcripts per million (TPM). Error bars represent standard deviation across biological replicates (N=2).

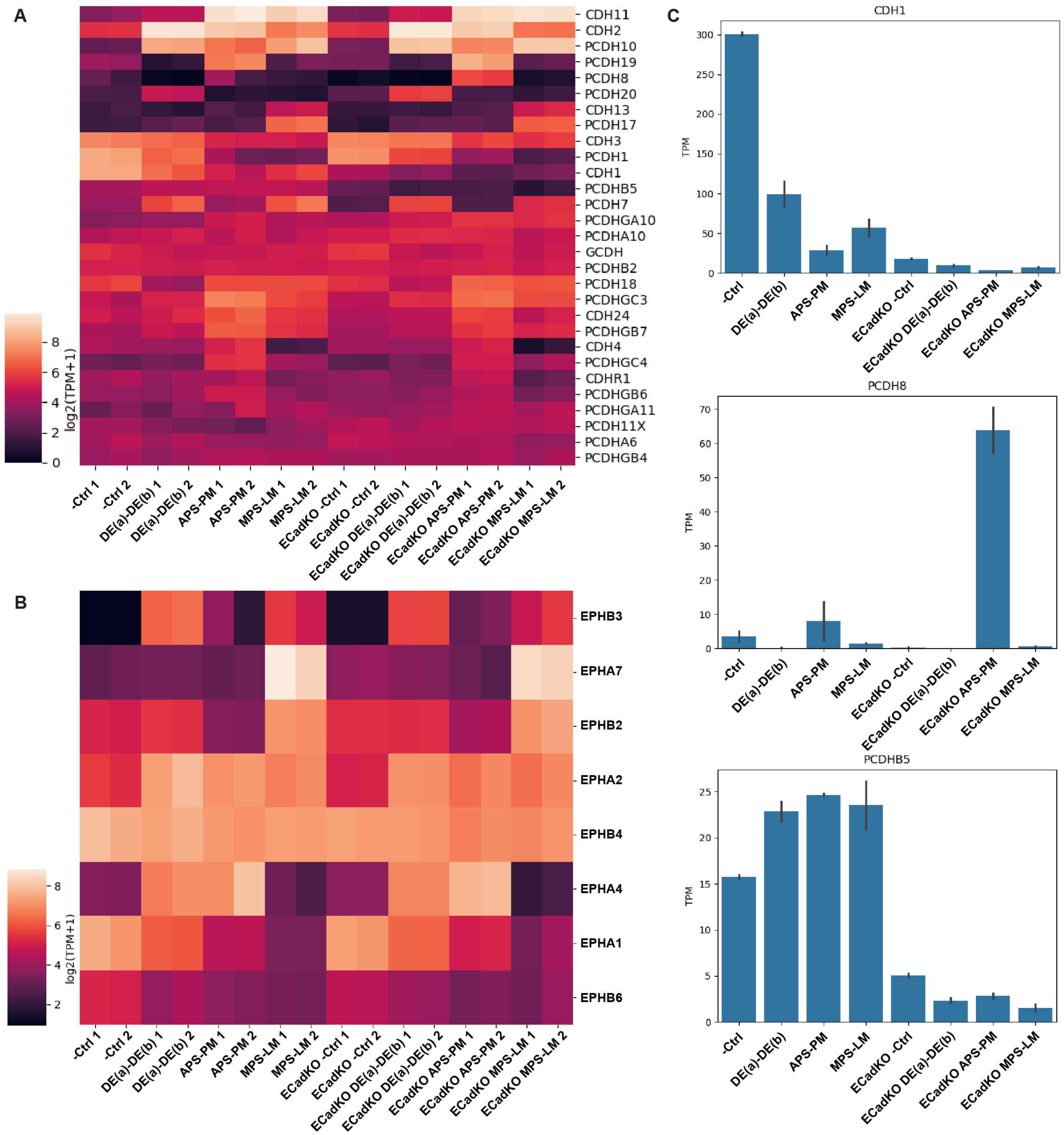

Fig. S21. Expression profiles of alternative cadherins in E-cadherin knockout cells. A, B. Heatmaps showing normalized transcript levels of classical cadherins (**A**) and ephrin family (**B**) across wild-type ESI017 and E-cadherin knockout cells under the indicated treatments. Data are presented as log2(TPM+1) from bulk RNA-seq. **C.** Bar plots of E-cadherin (CDH1), Protocadherin 8 (PCDH8), and Protocadherin beta-5 (PCDHB5) highlighting the absence of compensatory upregulation following CDH1 knockout. Error bars represent standard deviation across biological replicates (N=2).

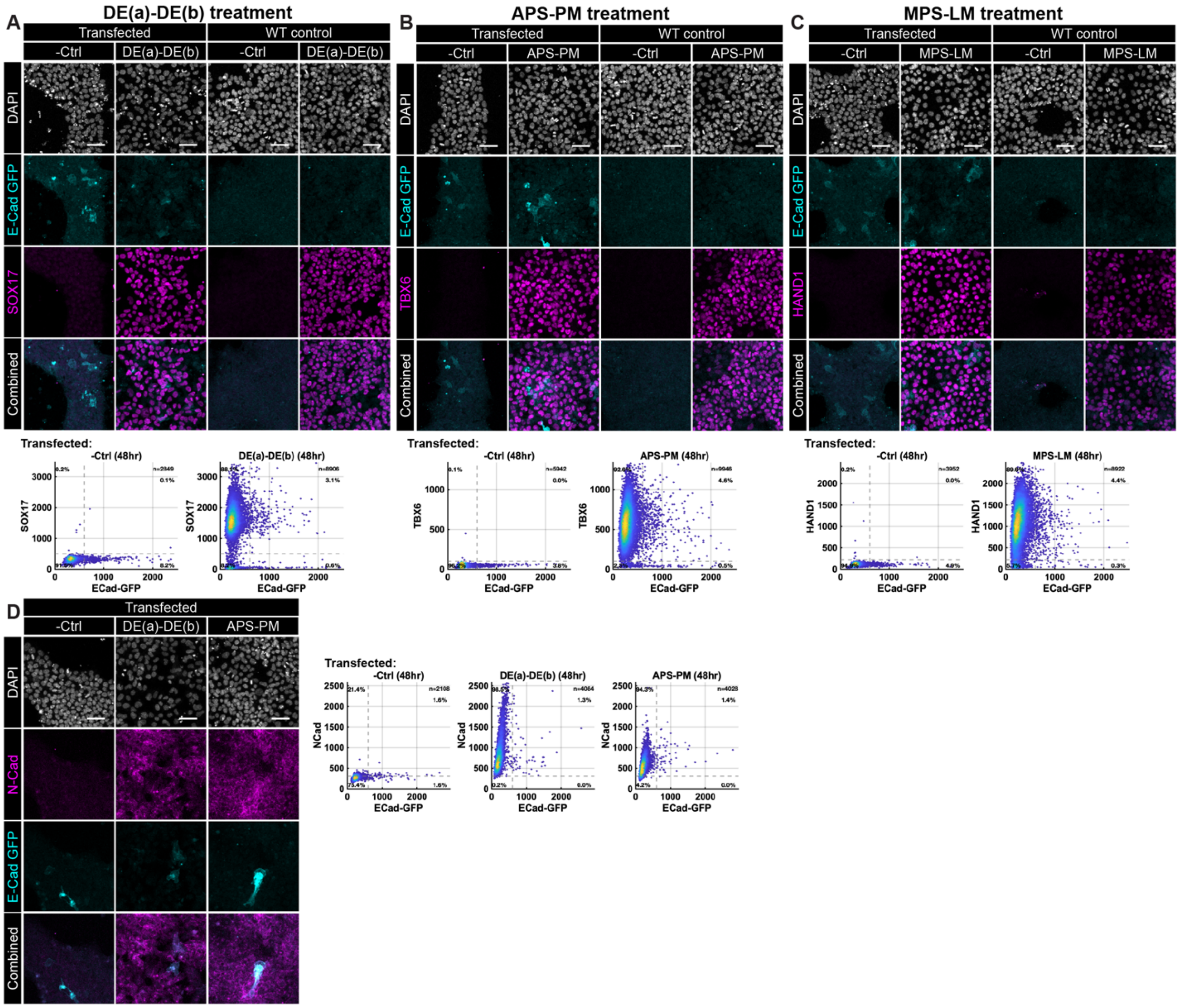

Fig. S22. Effects of E-cadherin over-expression on expression of lineage marker and N-cadherin. Example confocal immunofluorescent images immunostained for E-Cad-GFP, SOX17 (**A**), TBX6 (**B**), HAND1 (**C**), and N-Cad (**D**) for the indicated treatments. ESI017 cells were transfected using pcDNA3-E-cadherin-GFP construct for E-Cad over-expression induction before experiments. Scale Bars: 50 µm. Scatter plots show co-expression of the indicated markers at the single-cell level. Each dot represents one cell (n = [the total number of cells per condition]); color indicates density of overlapping points. Dashed lines denote manually defined thresholds used for quadrant separation. Percentages indicate the proportion of cells in each quadrant. (2 independent experiments performed. 10 images per condition.)

Development • Supplementary information

Table S1. Key Resource Table.

REAGENT or RESOURCE	SOURCE	IDENTIFIER
Multi-well plate for imaging		
μ-Plate 96 Well Round Black (with No. 1.5 polymer coverslip bottom)	Ibidi	Cat# 89606
μ-Slide 18 Well (with No. 1.5 polymer coverslip bottom)	Ibidi	Cat# 81816
Chemicals, Peptide, and Recombinant Proteins		
mTeSR1	Stemcell Technologies	Cat# 85875
mTeSR Plus	Stemcell Technologies	Cat# 100-0276
Matrigel	Corning	Cat# 354277
Dulbecco's PBS without calcium and magnesium	Caisson Labs	Cat# PBL01-6X500ML
Dispase	Stemcell Technologies	Cat# NC9886504
DMEM/F12	VWR	Cat# 45000-344
Accutase	Innovative Cell Technologies	Cat# NC9839010
Y-27632 dihydrochloride (ROCK inhibitor)	Tocris	Cat# 1254/10
Human recombinant laminin-521 protein	Biolamina	Cat# R021599/X0086842
Dulbecco's PBS with calcium and magnesium	Caisson Labs	Cat# PBL02-6X500ML
Essential 6 medium	Gibco	Cat# A15165-01
Recombinant Human/Mouse/Rat Activin A Protein	R&D Systems	Cat# 338-AC
CHIR99021	Medchemexpress LLC	Cat# 50-187-1892
Human FGF-basic (FGF-2/bFGF) (154 aa), Animal-Free Recombinant Protein	Gibco	Cat# AF-100-18B-100UG
Recombinant Human BMP-4 Protein	R&D Systems	Cat# 314-BP-050
LDN-193189	Stemolecule	Cat# 04-0074-02
A 83-01	Stemcell Technologies	Cat# 72022
IWP-2	Stemgent	Cat# 04-0034
Paraformaldehyde Aqueous Solution (PFA)	Electron Microscopy Science	Cat# 15710
Triton X-100	Sigma-Aldrich	Cat# 1001843780
Donkey Serum	Sigma-Aldrich	Cat# S30-100ML

Development • Supplementary information

Tween 20	Sigma-Aldrich	Cat# P1379
DAPI (4,6-diamidino-2-phenylindole, dihydrochloride)	Invitrogen	Cat# D1306
Clone R	Stemcell Technologies	Cat# 05889
Amaxa P3 Primary Cell 4D-Nucleofector X kit L	Lonza	Cat# V4XP-3012
Amaxa P3 Primary Cell 4D-Nucleofector X kit S	Lonza	Cat# V4XP-3032
TOPO™ TA Cloning™ Kit for Sequencing, without competent cells	Invitrogen	Cat# 450030
OneTaq Hot Start	NEB	Cat# M0489S
SpCas9 Nuclease	IDT	Cat# 1081061
Puromycin Dihydrochloride	Gibco	Cat# A1113803
Doxycycline hyclate	Sigma-Aldrich	Cat# D9891-1G
pcDNA3-E-cadherin-GFP construct	Addgene	plasmid #28009
Lipofectamine™ 3000 Transfection Reagent	Thermo Fisher Scientific	Cat# L3000015
Antibodies		
Rabbit Anti-E-Cad (1:300)	Cell Signaling Technology	Cat# 3195S
Mouse Anti-N-Cad (1:300)	Cell Signaling Technology	Cat# 14215S
Goat Anti-Snail (1:200)	R&D Systems	Cat# AF3639
Rabbit Anti-BRACHYURY (1:400)	R&D Systems	Cat# MAB20851
Mouse Anti-OCT3/4 (1:200)	BD Biosciences	Cat# 611203
Mouse Anti-FOXA2 (1:200)	BD Biosciences	Cat# 561580
Goat Anti-SOX17 (1:200)	R&D Systems	Cat# AF1924
Goat Anti-BRACHYURY (1:300)	R&D Systems	Cat# AF2085
Rabbit Anti-GATA3 (1:300)	Invitrogen	Cat# PA1-101
Mouse Anti-ISL1 (1:75)	Developmental Studies Hybridoma Bank	Antibody Registry ID# AB_2314683
Goat Anti-HAND1 (1:200)	R&D Systems	Cat# AF3168
Rabbit Anti-MIXL1 (1:300)	ABclonal	Cat# A17223
Mouse Anti-CDX2 (1:100)	Developmental Studies Hybridoma Bank	Antibody Registry ID# AB_2618482
Goat Anti-TBX6 (1:200)	R&D Systems	Cat# AF4744-SP

Development • Supplementary information

Rabbit Anti-SOX2 (1:200)	Cell Signaling Technologies	Cat# 5024S
Goat Anti-Nanog (1:400)	R&D Systems	Cat# AF1997
Goat Anti-GFP (1:400)	Rockland	Cat# 600-101-215s
Goat Anti-FOXF1 (1:200)	R&D Systems	Cat# AF4798
Rabbit Anti-Vimentin (1:300)	Cell Signaling Technology	Cat# 5741
Rabbit Anti-ZO-1 (1:300)	Cell Signaling Technology	Cat# 5406S
Mouse Anti-EpCam (1:300)	Cell Signaling Technology	Cat# 2929T
Donkey anti-Rabbit IgG (H+L) Highly Cross-Adsorbed Secondary Antibody, Alexa Fluor™ 488	Invitrogen	Cat# A-21206
Donkey anti-Mouse IgG (H+L) Highly Cross-Adsorbed Secondary Antibody, Alexa Fluor™ 555	Invitrogen	Cat# A-31570
Donkey anti-Goat IgG (H+L) Cross-Adsorbed Secondary Antibody, Alexa Fluor™ 647	Invitrogen	Cat# A-21447
RNAqueous™-Micro Total RNA Isolation Kit	Invitrogen	Cat# AM1931
Experimental Models: Cell Lines		
ESI-017	ESI BIO	RRID:CVCL_B854
ESI-017 CAAX-mCFP (dox inducible)		
ESI-017 E-Cad:mCitrine - N-Cad:mCherry-CAAX:mCerulean(dox inducible)		
ESI-017 E-Cad $^{-/-}$		
Software and Algorithms		
MATLAB		https://www.mathworks.com/products/matlab.html
ilastik	(Berg et al., 2019)	http://ilastik.org/
FIJI	(Schindelin et al., 2012)	https://fiji.sc/
TIDE	(Brinkman et al., 2014)	http://shinyapps.datacurators.nl/tide/
Salmon	(Patro et al., 2017)	

Development • Supplementary information

Table S2. Primers for genomic DNA PCR

Target Gene	Forward	Reverse
CDH2	5' AAGGCAGTGGCTCCACTGC 3'	5' ACTGATATTCCCTCTGAGCCC 3'
CDH2-mCherry-HDR	5' CCACGGTTCAAGAAACTTGCTGACATGTATGGTGGAGGTGATGACATGgtgagcaagggc 3'	5' ATTGTTTGTACTTGTCCAAAAACCAAGTTCACCCTGAAGTTCAacggtgcctgGGATCCg 3'
HindIII-mCherry		5' AAGCTTcttgtacagctcgtccatgcc 3'
Hind-loxp-EcoR	5' AGCTTATAACTTCGTATAGCATACATTATACGAAGTTATG 3'	
NeoR	5' AACTGCAGGACGAGGCAGC 3'	
polyA	5' ATGCCTGCTCTTTACTGAAGG 3'	
CDH1-EXON3	5'GTTACAGGCATGAGACACTGT 3'	5'ATAAGGAAGCTCAAGCATAGAC3'

Table S3. Lists of the top 50 upregulated and downregulated genes by ECAD-KO in control, DE, PM, and LM differentiation. Treatment is indicated in the sheet name. Expression values given are log2(TPM+1).

Available for download at
https://journals.biologists.com/dev/article-lookup/doi/10.1242/dev.204807#supplementary-data

Table S4. Lists of the top 20 GO terms associated with the genes affected by loss of ECAD in control, DE, PM, and LM differentiation. Treatment and direction of regulation is indicated in the sheet name.

Available for download at
https://journals.biologists.com/dev/article-lookup/doi/10.1242/dev.204807#supplementary-data

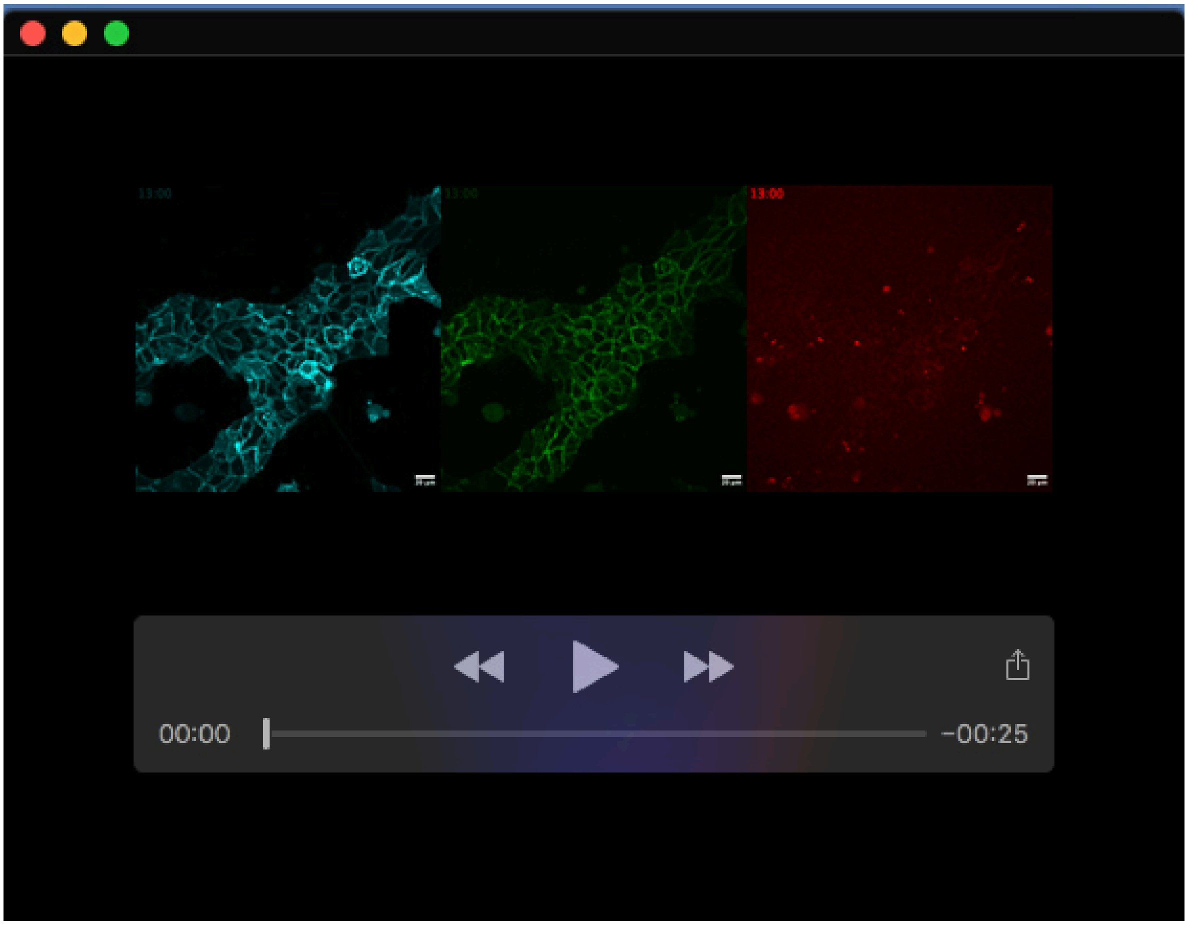

Movie 1. Timelapse movie of ESI017-CDH1:mCitrine-CDH2:mCherry-CAAX:mCerulean reporter cell line for 44.5 hours of MPS-LM treatment. Live imaging was performed using confocal microscopy. Cell membrane is shown in cyan (mCerulean-CAAX), CDH1 (E-cadherin) in green (mCitrine), and CDH2 (N- cadherin) in red (mCherry), and. Scale bar: 20 µm.
